# When Pleasure Painted the Blood Blue: A Case of Popper-Induced Methemoglobinemia

**DOI:** 10.7759/cureus.89694

**Published:** 2025-08-09

**Authors:** Abir Aijaz, Abdul Bhat, Hadiya Chisti, Vignesh S Jeevaraj, Muhammad Hamid

**Affiliations:** 1 Acute and General Internal Medicine, Weston General Hospital, University Hospitals Bristol and Weston, Weston-super-Mare, GBR; 2 Acute Medicine, Weston General Hospital, University Hospitals Bristol and Weston, Weston-super-Mare, GBR; 3 Medicine, Weston General Hospital, University Hospitals Bristol and Weston, Weston-super-Mare, GBR; 4 Emergency Medicine, Weston General Hospital, University Hospitals Bristol and Weston, Weston-super-Mare, GBR

**Keywords:** collapse, inhalation, methemoglobin, peripheral cyanosis, poppers

## Abstract

Methemoglobinemia is an uncommon yet potentially life-threatening condition that results from the oxidation of iron from the ferrous (Fe²⁺) to the ferric (Fe³⁺) state, rendering hemoglobin unable to effectively transport oxygen. This translates into a state of functional hypoxia despite adequate arterial oxygen tension. Among the various causes of acquired methemoglobinemia, recreational inhalation of alkyl nitrites, widely known as “poppers,” is a notable but underrecognized trigger. Timely diagnosis and treatment are essential, as the condition can rapidly deteriorate without intervention.

We describe the case of a 59-year-old man who presented to the emergency department with collapse, confusion, and prominent central cyanosis following the inhalation of poppers. Interestingly, his pulse oximetry readings were within the normal range, registering between 95 and 98% on room air, which was incongruent with his clinical appearance. This prompted a thorough evaluation, including arterial blood gas analysis, which revealed a markedly elevated methemoglobin level of 29%. Routine blood tests were otherwise unremarkable, and no other contributing toxic exposures were identified. Management involved immediate administration of high-flow supplemental oxygen and intravenous methylene blue at a dose of 1 mg/kg. The patient responded rapidly with the resolution of cyanosis and restoration of normal mental status. Follow-up testing demonstrated a significant decline in methemoglobin levels, and the patient was discharged in full recovery with no residual complications.

This case highlights the diagnostic challenges associated with methemoglobinemia, particularly as standard pulse oximetry is unreliable in detecting dyshemoglobinemias and often fails to reflect the true degree of tissue hypoxia. Clinicians should maintain a high index of suspicion when faced with unexplained cyanosis, especially in the context of normal PaO₂ values and possible recreational drug use. Methylene blue remains the gold-standard antidote, functioning as an electron donor that facilitates the reduction of methemoglobin to its oxygen-carrying ferrous state.

Early recognition and prompt treatment of methemoglobinemia are vital for preventing serious morbidity or mortality. Moreover, public awareness campaigns addressing the potential toxicological consequences of poppers use are essential to reduce the recurrence of such preventable emergencies. This case underscores the need for emergency physicians and frontline clinicians to consider methemoglobinemia in the differential diagnosis of collapse, unexplained cyanosis, or altered mental status, even when conventional oxygen measurements appear reassuring.

## Introduction

Methemoglobinemia is an underdiagnosed, rare disorder with an incidence of approximately 1 in 10,000 individuals [1]. It may partly be congenital due to enzyme deficiencies or acquired due to exposure to oxidant drugs and chemicals such as nitrates, nitrites, sulfonamides, and certain anesthetics [2]. Recreational use of alkyl nitrites, known as “poppers,” has increasingly been associated with cases of acquired methemoglobinemia [3]. Poppers or volatile alkyl nitrites are inhaled for their vasodilatory and euphoric effects by adolescents usually. They have a capability to oxidize hemoglobin to methemoglobin, reducing the ability of red blood cells to transport and carry oxygen [4]. This report earmarks a case of acute methemoglobinemia precipitated by poppers use.

## Case presentation

**Figure 1:**
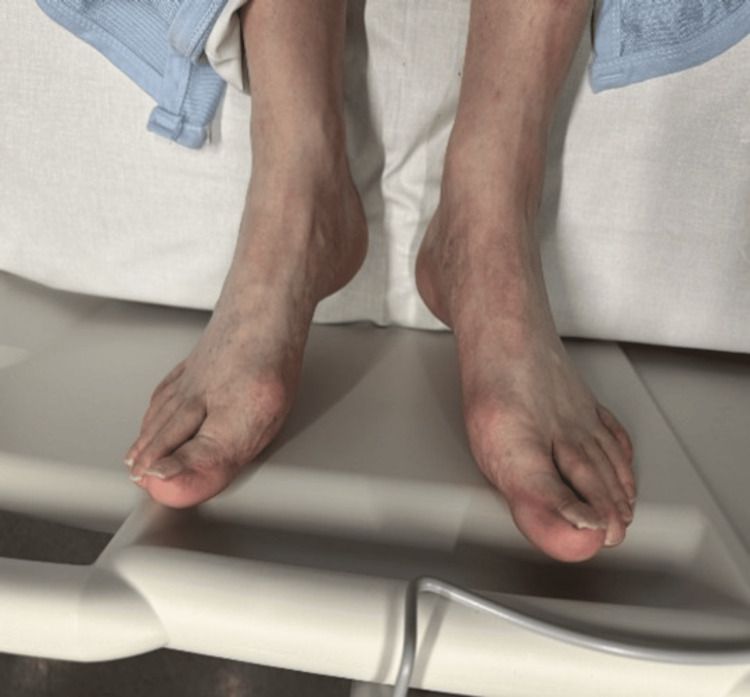
Bluish hue noted in feet

A 59-year-old Caucasian man with nil acute past medical history presented to the emergency department with a two-day history of global headache, followed by collapse at a bar where he was partying. Bystanders reported that he had been sniffing poppers prior to the incident. Emergency services were called in and found him with a Glasgow Coma Scale (GCS) score of 6. Upon transport to the hospital, his GCS improved to 15 with basic supportive measures like giving supplemental oxygen, although he remained slightly confused. On presentation to the hospital, he was cyanotic (Figure 1) and slightly tachycardic, and his oxygen saturation was normal with nil acute systemic examination otherwise except for purplish discoloration of fingertips and peripheries. The rest of his systemic examination was normal.

His arterial blood gas (ABG) revealed significantly high methemoglobin levels of 29 percent (reference range - < 1.5 percent) with normal oxygen saturation and PaO2 levels (Table 1).

**Table 1:** Laboratory values

Lab Parameter	Value	Range
pH	7.36	7.35-7.45
PaO2	10.6	8.8-12 KPA
PCO2	5.6	4-6 KPA
Meth HB	29 Percent	<2
Bicarbonate	26.4	22-28 meq/l

**Figure 2:**
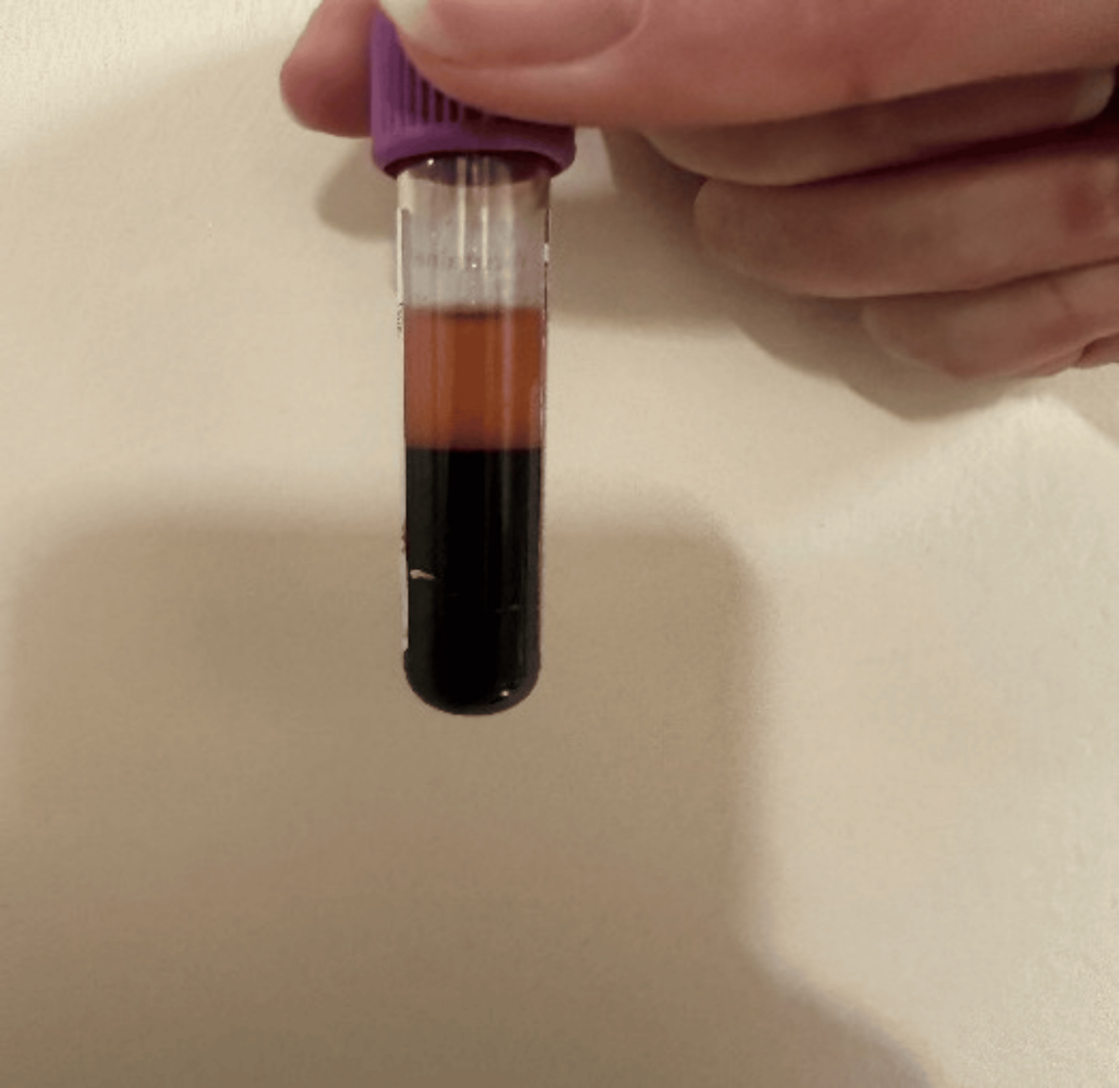
Chocolate-brown colored blood

Given the patient’s acute presentation with altered mental status and cyanosis, several differential diagnoses were initially considered. Traumatic brain injury was excluded due to the absence of any history of trauma and normal findings on neurological imaging. Alcohol intoxication was also ruled out based on the clinical picture and negative toxicology screening. Anoxic brain injury was deemed unlikely given the patient’s prompt neurological recovery and normal ABG oxygenation parameters. Ultimately, drug-induced methemoglobinemia emerged as the leading diagnosis, supported by the characteristic clinical features, significantly elevated methemoglobin levels, and a clear history of inhalation of poppers a known precipitant of this condition. Most of his laboratory studies were normal including a complete blood count barring chocolate-brown colored blood (Figure 2), renal function, and liver enzymes. Toxicology screens were negative for ethanol, opioids, and amphetamines.

The patient was started on high-flow oxygen via a non-rebreather mask and was given methylene blue 1 mg/kg diluted in 100 mL of 5% dextrose over five minutes. Within half an hour, his cyanosis improved, and confusion settled. Subsequent ABG revealed a methemoglobin level of 3%. The patient was successfully discharged the following day with advice to avoid all precipitants, especially alkyl nitrites. He was offered toxicology counseling and provided with educational materials including pamphlets on the potential dangers and hazards of recreational drug use.

## Discussion

This case illustrates a classic presentation of popper-induced methemoglobinemia, a rare but potentially life-threatening diagnosis that should be considered in patients with unexplained cyanosis and normal pulse oximetry readings [5]. Pulse oximetry can be misleading in such cases because it cannot differentiate oxyhemoglobin from methemoglobin, often producing stable values around 85-90% regardless of true oxygenation [6]. This can delay recognition, highlighting the need for co-oximetry via ABG analysis, which remains the gold standard for diagnosis.

Poppers, including alkyl nitrites such as amyl nitrite and isobutyl nitrite, are powerful oxidizing agents that convert hemoglobin’s iron from the ferrous (Fe²⁺) to the ferric (Fe³⁺) state, forming methemoglobin [7]. Methemoglobin cannot effectively bind or transport oxygen, leading to functional hypoxia. Additionally, its presence induces a leftward shift in the oxygen-hemoglobin dissociation curve, further limiting oxygen delivery to tissues [8]. Together, these mechanisms explain the rapid onset of cyanosis, dyspnea, and altered mental status in affected patients.

Clinically, methemoglobinemia presents with features such as central cyanosis, shortness of breath, confusion, and chocolate-brown colored blood that does not turn red when exposed to air [9]. A lack of response to high-flow oxygen therapy is a hallmark of this condition. Diagnosis should be considered when there is a mismatch between the patient’s clinical appearance and the oxygen saturation indicated by pulse oximetry.

Treatment depends on severity. Mild cases, with methemoglobin levels below 20% and minimal symptoms, often resolve with discontinuation of the offending agent and supplemental oxygen. Moderate to severe cases, especially when levels exceed 20-30%, require methylene blue, which serves as an electron donor for NADPH methemoglobin reductase, facilitating the conversion of methemoglobin to functional hemoglobin [10]. However, methylene blue is contraindicated in patients with glucose-6-phosphate dehydrogenase (G6PD) deficiency due to the risk of hemolysis, and alternatives such as high-dose ascorbic acid, exchange transfusion, or hyperbaric oxygen may be considered [11].

The rising incidence of popper-related methemoglobinemia reflects the increased recreational use of alkyl nitrites, which are widely available and often marketed as room deodorizers or cleaning products. Their perceived harmlessness and easy accessibility contribute to unintentional toxicity, particularly among adolescents and young adults [6]. Additionally, the social stigma surrounding recreational drug use may prevent patients from disclosing their exposure, complicating timely diagnosis and treatment. Therefore, clinicians must maintain a high index of suspicion, especially in patients with unexplained cyanosis, normal arterial oxygen tension (PaO₂), and pulse oximetry values inconsistent with clinical findings.

Awareness and education are essential in preventing such cases. Public health initiatives should aim to increase knowledge about the potential toxicity of alkyl nitrites, while healthcare professionals should be trained to recognize the subtle diagnostic clues of methemoglobinemia. Simple measures, such as asking about recreational drug use during emergency evaluations and recognizing the characteristic chocolate-colored blood, can lead to earlier intervention and improved outcomes.

This case reinforces several important clinical lessons. First, unexplained cyanosis with normal or near-normal oxygen saturation readings should prompt consideration of dyshemoglobinemias such as methemoglobinemia. Second, co-oximetry remains the diagnostic test of choice, as standard pulse oximetry fails to detect abnormal hemoglobin species. Third, while methylene blue remains the treatment cornerstone, its use requires caution in individuals with G6PD deficiency, necessitating alternative strategies when contraindicated. Finally, the growing recreational use of poppers underscores the importance of preventive strategies, including public education and regulation.

## Conclusions

Popper-induced methemoglobinemia, although uncommon, is a critical toxicological emergency. Prompt recognition and treatment are vital, as delayed management can lead to severe tissue hypoxia and fatal outcomes. Clinicians should remain vigilant when faced with cyanosis unresponsive to oxygen therapy, particularly in the context of recreational drug use. Early diagnosis with co-oximetry and prompt administration of methylene blue can lead to rapid recovery, as demonstrated in this case. Increased awareness among both healthcare professionals and the public is key to reducing the morbidity and mortality associated with this preventable condition.
